# Morphometrical and molecular identification of *Echinococcus granulosus* genotypes in wild canids in north of Iran

**DOI:** 10.1002/vms3.1554

**Published:** 2024-08-08

**Authors:** Moein Abolhasani Darounkola, Elahe Ebrahimzadeh, Hassan Borji, Mohammadreza Khoshvaght

**Affiliations:** ^1^ Department of Pathobiology Faculty of Veterinary Medicine Ferdowsi University of Mashhad Mashhad Iran

**Keywords:** *Echinococcus granulosus*, genotypes, Iran, molecular identification, wild canids

## Abstract

**Background/Objective:**

The cestode *Echinococcus granulosus* causes cystic echinococcosis, a zoonotic parasitic infection that constitutes a significant public health risk. This parasite has been documented to have potential reservoirs and carriers among wild canids, namely wolves, foxes and jackals. This study aimed to determine the prevalence and molecular characteristics of *E. granulosus sensu lato* species/genotypes among wild canids in three northern, northeastern and north‐western Iran regions.

**Methods:**

From 2019 to 2022, 93 wild canid carcasses (69 jackals), (22 foxes) and (2 wolves) were collected that were killed in car accidents or illnesses. Analyses of morphology and morphometry were performed to verify the presence of *E. granulosus*. To determine *E. granulosus s.l*. species/genotypes, polymerase chain reaction (PCR)‐RFLP (ITS1) was performed utilizing the Bsh1236I (BstUI) restriction enzyme. *COX1*, *NADH1* and *ITS1* gene sequencing were also performed to confirm the PCR‐RFLP results.

**Results:**

During this study, 93 wild canids were examined, and 3.2% (95% CI: 0%–7%) of the 93 were infected with *Echinococcus*. The north‐western region of Iran showed two out of 30 jackals (6.6%) infected with adult *Echinococcus* compared to one out of 35 jackals (2.8%) in the northern region. DNA from *Echinococcus* was detected in these individuals by PCR. Based on PCR‐RFLP analysis of the *ITS1* gene and sequencing of *COX1*, *NADH1* and *ITS1* gene, *E. granulosus sensu stricto* genotype was confirmed in the jackals that had been infected.

**Conclusion:**

Evidence shows that *E. granulosus* occurs in jackals in Iran, with the *E. granulosus s.s*. genotype being the most common. This parasite has been identified as a zoonotic parasite with a genotype that can be transmitted to livestock and humans. Establishing effective control measures to prevent the spread of echinococcosis and ensure public health is crucial.

## Graphical abstract

This study reveals minor genetic variations and high genetic similarity among the *Echinococcus granulosus* populations in Iran and other countries, indicating a global genetic connectivity and international dispersion of this parasite. Therefore, global cooperation is essential for its control. Dominant *Echinococcus* species based on morphology and morphometric characterization was identified in golden Jackal in this project was *E. granulosus*. The dominant *E. granulosus* genotype was identified in golden Jackal in this project was G1.

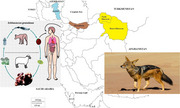

## 1 INTRODUCTION

It is well known that *Echinococcus granulosus sensu lato* (*s.l*.), the agent of cystic echinococcosis (CE), poses a significant health risk to humans and animals (Alvi et al., 2023; Vuitton et al., 2020). The disease is of significant economic importance in many developing countries. The economic cost of CE in Iran was estimated at US$232.3 million per year (Fasihi Harandi et al., 2012). Recent molecular investigations have detected that *E. granulosus* comprises a complex species known as *E. granulosus s.l*. This complex includes *E. granulosus sensu stricto (s.s.)* (G1–G3), *Echinococcus equinus* (G4), *Echinococcus ortleppi* (G5), *Elodea canadensis* (G8, G10), *Euoniticellus intermedius* (G6, G7) and *Echinococcus felidis* (Casulli et al., 2022; Hua et al., 2022; Laurimäe, Kinkar, Moks, et al., 2018; Romig et al., 2015; Thompson, 2020). Genotypes are separated based on several criteria, such as epidemiology and geographic distribution, intermediate host spectrum, adult and metacestode morphology, maturation time in definitive hosts, the tissue tropism of larval stages and pathogenicity in humans (Beyhan et al., 2020; Casulli et al., 2022; Han et al., 2019; Maldonado et al., 2017; Muqaddas et al., 2023; Santolamazza et al., 2020).

*E. granulosus s.s*. (G1–G3) has mainly a domestic life cycle between dogs as a final host and livestock as an intermediate host. However, some regions have reported wildlife transmission (Hüttner et al., 2009; Otero‐Abad & Torgerson, 2013). It is important to pay attention to this point; intermediate hosts such as sheep can be killed and hunted by wild carnivores, and the sylvatic transmission cannot be ignored (Kagendo et al., 2014).

Dogs, wolves, foxes and jackals are some of the frequently affected canid species (Tamarozzi et al., 2020). Intermediate hosts such as livestock and humans can risk getting infected with *E. granulosus s.l*. by infected carnivores, consuming eggs from contaminated foods or drinking water (Grakh et al., 2020; Pal et al., 2022). Larvae land inside the body and evolve into a cyst, which often takes place in the lungs and liver (Gessese, 2020). Wildlife reservoirs are animals that can host parasites and facilitate their maintenance and transmission (Wells & Flynn, 2022). A complete understanding of the parasite's dynamics, ecology and potential hazard to public health depends on the identification and study of its reservoirs in wildlife (Jenkins, 2021; Laurimäe, Kinkar, Romig, et al., 2018). There are, however, several factors that make research in this area challenging. It is also logistically tricky and demands considerable resources to conduct surveillance and sampling in wildlife populations.

In evaluating the level of environmental contamination and investigating the transmission dynamics of echinococcosis, a significant factor that should be considered is the prevalence of adult *E. granulosus s.l*. in canid wild populations (Harriott et al., 2019). Depending on the geographical region of Iran, the prevalence of CE in stray dogs was reported between 7% and 64%, and in owned dog between 3% and 63% (Borhani et al., 2021). Limited studies have focused on *E. granulosus s.l*. in wild carnivore species in Iran. *E. granulosus s.l*. was found in the faecal samples of 16.9% of 77 dogs, 66.7% of 9 jackals, and all of the foxes, the wolf and the hyena (3 foxes and 1 wolf) (Beiromvand et al., 2011). Another study was found 6.6% of the canines (four dogs, two jackals and one wolf) to be infected with *granulosus s.s*. (G1) (Heidari et al., 2019).

According to a recent meta‐analysis in Iran, the pooled prevalence of CE in human beings was 5% (Ahmadi & Meshkehkar, 2011; Borhani et al., 2021; Eslami et al., 2014; Mahmoudi et al., 2019). Based on recent studies, the prevalence of livestock CE in Iran ranges from 1.3% to 74.4% in sheep, 0.4% to 37.8% in goats, 4.3% to 31.9% in buffaloes, 1.3% to 40.1% in cattle, 8.8% to 40.4% in camels and 2% in donkeys (Ahmadi, 2005; Borhani et al., 2021). The most common strain in livestock in Iran was *E. granulosus s.s*. (G1, G3) and G6 (Borhani et al., 2021).

In Iran and worldwide, studies of hydatid cyst prevalence have primarily focused on intermediate hosts, and information on wildlife populations needs to be included (Khademvatan et al., 2019; Parkoohi et al., 2018). Much importance is attributed to identifying and comprehending *E. granulosus s.l*. species/genotypes in the final hosts and wild canids worldwide (Gessese, 2020). An in‐depth knowledge of the various *E. granulosus s.l*. species/genotypes is essential. Based on molecular identification and the geographical distribution of *E. granulosus* species/genotypes, scientists can develop specialized control measures against the disease. Comprehending epidemiology and designing effective surveillance strategies by distinguishing strains in distinct regions is also helpful. Accordingly, this study was intended to investigate the prevalence of *Echinococcus* infection in wild canids in some regions of Iran based on morphological and morphometrical study. Furthermore, this study aimed to evaluate the cestode's phylogenetic analysis to determine *E. granulosus s.l*. species/genotypes.

## 2 MATERIALS AND METHODS

### 2.1 Study regions

In this study, three regions have been chosen based on their diverse weather conditions and geographical aspects of this research. Iran's northern, northwest and northeastern provinces were selected. While maintaining its varied features, each region has a unique climate.

The northern area of Iran (36°19′15′′ N 52°37′58′′ E), located along the Caspian Sea, is characterized by a humid and mild climate. This region is characterized by dense forests and abundant vegetation, making it an ideal habitat for various wildlife species, including foxes, jackals and wolves. The presence of these canid species in the northern region makes studying the prevalence of *Echinococcus* infection in this area worthwhile.

Iran's north‐western region (38°125′13′′ N 47°42′17′′ E) is adjacent to Turkey and Armenia. This region's climates range from semi‐arid to arid, including mountains, plateaus and valleys.

**FIGURE 1:**
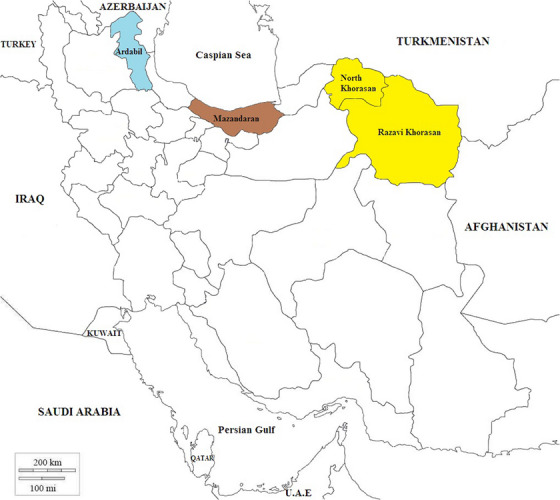
Map of Iran, geographical locations of north‐east (Razavi Khorasan and North Khorasan Provinces), north (Mazandaran Province) and north‐west (Ardabil Province).

In the northeastern part of the country, which borders Afghanistan and Turkmenistan, vast deserts, plateaus and mountain chains exist. In this region, the climate is primarily semi‐arid and arid, whereas the ecosystems of deserts and semi‐deserts are exceptional. This area is home to various wildlife species, including wild canids. Moreover, studies have yet to be done among neighbouring countries, allowing us to gain a better knowledge of *E. granulosus s.l*. species/genotypes, which also exist there (Figure 1).

### 2.2 Sample collection

Between 2019 and 2022, 93 carcasses of jackals, foxes and wolves that died in road accidents were collected in three regions in Iran. The intestines were isolated from each carcass, ligatured and preserved in 70% ethanol. During the study, proper precautions were taken to safeguard the researchers’ health and safety. All procedures were conducted according to the code of ethics (Ethical code: IR.UM.REC.1401.108).

Under safe conditions, a technique known as Intestinal Scraping Technique was applied to examine the small intestines for *Echinococcus* (Eckert & Deplazes, 2004). The mucosa was meticulously scraped using a laboratory spatula after the intestine was longitudinally opened with scissors. After washing the substance onto a sieve, *Echinococcus* was separated from the contents with a stereomicroscope. Until further tests could be performed on the obtained specimens, the collected samples were preserved in 70% ethanol.

### 2.3 Morphological and morphometric examination

Several factors were taken into account to determine the worms’ morphology, including their length, their anterior part to the gravid proglottid, how many scolex hooks they had, how large and small their hooks were (hook length, hook width, blade length and width, handle length and width), the shape of the uterus and the location of the genital pore. To do this, digital images of the samples were captured at several magnification levels via a calibrated digital camera (Genius IS500). Polyvinyl alcohol was applied to the glass slide that contained adult scolexes for microscopic analysis. TCapture software was employed to process the captured images. This study used valid identification keys and the mentioned characteristics to identify adult worms (Thompson et al., 1984). A carmine acid staining method enabled us to visualize uterine branches and other morphological features (Dubinský et al., 1998).

After morphological and morphometrical examinations of all sampled worms, a more comprehensive analysis was conducted using the polymerase chain reaction (PCR) technique on the positive samples.

### 2.4 Molecular analysis

#### 2.4.1 DNA extraction

Genomic DNA was extracted from all samples, which were morphologically diagnosed using a DNA extraction kit (MBST). The DNA samples were then used to molecularly confirm the identity of the *Echinococcus* specimens that were previously morphologically identified from each tapeworm. To preserve the extracted DNA for later usage, a temperature of −20°C was maintained for storage.

#### 2.4.2 PCR

##### PCR (ITS1 gene)

*ITS‐1*‐forward (5′‐ATG GTT GTT ATC GCT GCG A‐3′) and *ITS‐1*‐reverse (5′‐CAG AGC ACT TTT GTA TGC A‐3′) comprise the first set of primers. The PCR reaction mixture (25 µL) consisted of 2× Master mix (12.5 µL), each primer (1 µL, 10 µM), template DNA (2 µL, ∼100 ng) and dH_2_O (8.5 µL). Thirty‐five cycles of PCR were conducted, consisting of 5 min of initial denaturation at 94°C, 45 s of denaturation at 95°C, 45 s of annealing at 50°C and 45 s of extension at 72°C. After a final extension step of 10 min at 72°C, the PCR process was completed. PCR experiments included negative and positive controls with distilled water and *Echinococcus* DNA, respectively. As part of the PCR analysis, the amplified products were electrophoresed on 1.5% agarose gels, separated in tris‐borate‐EDTA (TBE) buffer, stained with Safe Stain and visualized through a UV illuminator.

An ITS1 PCR‐RFLP analysis was performed to determine *E. granulosus s.l*. species/genotypes. In the following steps, 10 µL of the PCR amplicons were digested with 1 µL Bsh1236I (Fermentas) with 2 µL 10× Buffer R and 7 µL nuclease‐free water, in a final 20 µL volume and incubated for 7 h at 37°C. In the final stage, ITS1 PCR‐RFLP product was electrophoresed on 3% agarose gels and stained with Safe Stain to distinguish the digested fragments.

##### PCR (cytochrome oxidase subunit I gene)

Amplification of the cytochrome oxidase subunit I (*COXI*) gene was achieved through the first primer pairs *JB3* (5′‐TTT TTT GGG CAT CCT GAG GTT TAT‐3′) and *JB4.5* (5′‐TAA AGA AAG AAC ATA ATG AAA ATG‐3′) (Bowles et al., 1992). PCR experiments were performed in a combined volume of 25 µL, including 12.5 µL of 2× Master mix, 1 µL of each primer (10 µM), 2 µL of template DNA (approximately 100 ng) and 8.5 µL of dH_2_O. PCR protocols included 94°C for 5 min for initial denaturation, followed by 38 cycles, 30 s at 94°C for denaturation, 45 s at 50°C for annealing, 45 s at 72°C for extension and 10 min for final extension. Each PCR reaction included distilled water negative controls and *Echinococcus* DNA positive. The PCR products were electrophoresed on 1.5% agarose gels, separated in TBE buffer, stained with Safe Stain, and visualized through a UV illuminator (Bowles et al., 1992).

##### PCR (NADH subunit 1 gene)

Moreover, PCR experiments were used with the second primer pair *JB11* (5′‐AGA TTC GTA AGG GGC CTA ATA‐3′) and *JB12* (5′‐ACC ACT AAC TAATTC ACT TTC‐3′), which targeted the gene for the NADH dehydrogenase subunit 1 (*NADH1*) gene of *Echinococcus* (Bowles & McManus, 1993). The PCR method was performed as described in the previous section for *COXI*.

#### 2.4.3 Sequencing

Two‐way sequencing (BGI) was performed using PCR products derived from each gene (*ITSI*, *COXI* and *NADH1*) on samples from diverse morphological and geographical regions. A comparison was made between the nucleotide sequences obtained from the *ITSI gene*, *COXI* gene and *NADH1* gene and those from GenBank.

#### 2.4.4 Phylogenetic analysis

A phylogenetic tree of *E. granulosus COXI* and *NADH1* gene retrieved from north and north‐western Iran (in this study) and reference sequences deposited in GenBank related to other *E. granulosus s.l*. species/genotypes constructed with MEGA 11 software, applying the neighbour‐Joining method with bootstrap analysis (500 replicates). *Taenia solium* was chosen as the outgroup.

As well as, phylogenetic networks were calculated based on 460 bp of the CoxI gene and 540 bp of the NADH1 gene. Seventeen datasets were used to compare the phylogenetic power of gene fragments to differentiate genotypes G1–G10. Network analyses were calculated using https://www.fluxus‐engineering.com/sharenet.htm (Abdullah et al., 2022).

### 2.5 Statistical analysis

This study conducted both Kolmogorov–Smirnov and Shapiro–Wilk tests to assess the distribution of the datasets, which is essential for selecting appropriate statistical analyses. The data analysis regarding the mean length of the worm and mean size of hooks was performed using SPSS software version 21 for all analyses. The statistical significance ratio was set as *p* < 0.05.

## 3 RESULTS

### 3.1 Prevalence

There were 42 *Echinococcus* tapeworms collected from 93 carcasses. In the northeastern region, there were a total of 28 carcasses, including 17 jackals (8 females and 9 males), 9 foxes (6 females and 3 males) and 2 wolves (1 female and 1 male). In the northern region, there were a total of 35 jackals, comprising 22 females and 13 males, with a reported total of 35 carcasses. In the north‐western region, there were a total of 17 jackals, with 10 females and 7 males, and 13 foxes, with 6 females and 7 males.

During this study, three wild canids, 3.2% (95% CI: 0%–7%) of the 93 examined were infected with *Echinococcus* tapeworms. Study results revealed no evidence of *Echinococcus* infection in foxes and wolves. The north‐western region of Iran (Ardabil province) showed 2 out of 30 jackals (6.6%) infected with adult *Echinococcus* tapeworms when compared with one out of 35 jackals (2.8%) in the northern region (Mazandaran province). No infections with this parasite were observed in the northeastern region (Khorasan Razavi and North Khorasan provinces).

### 3.2 Morphological and morphometric examination

**FIGURE 2:**
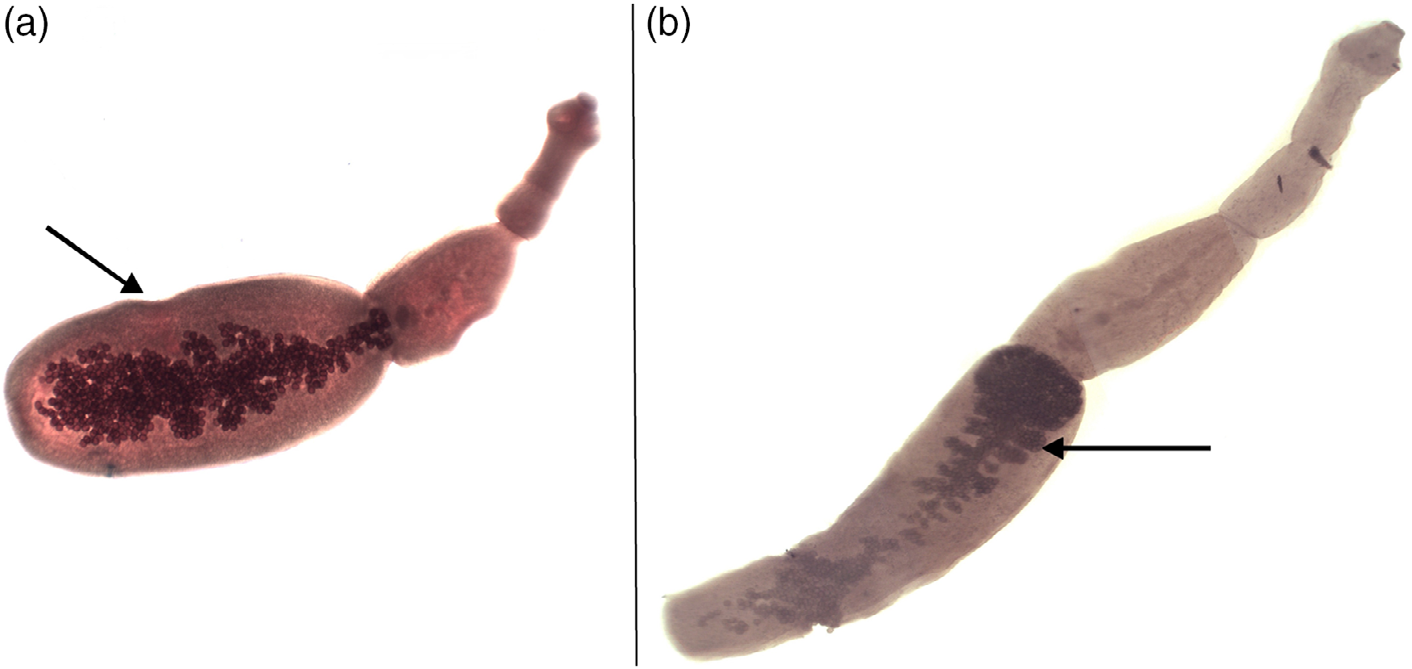
Morphological appearance of *Echinococcus granulosus* adult worm from north of Iran (a) and northwest of Iran (b) the uterus containing the eggs is indicated by the arrow: (a and b).

**FIGURE 3:**
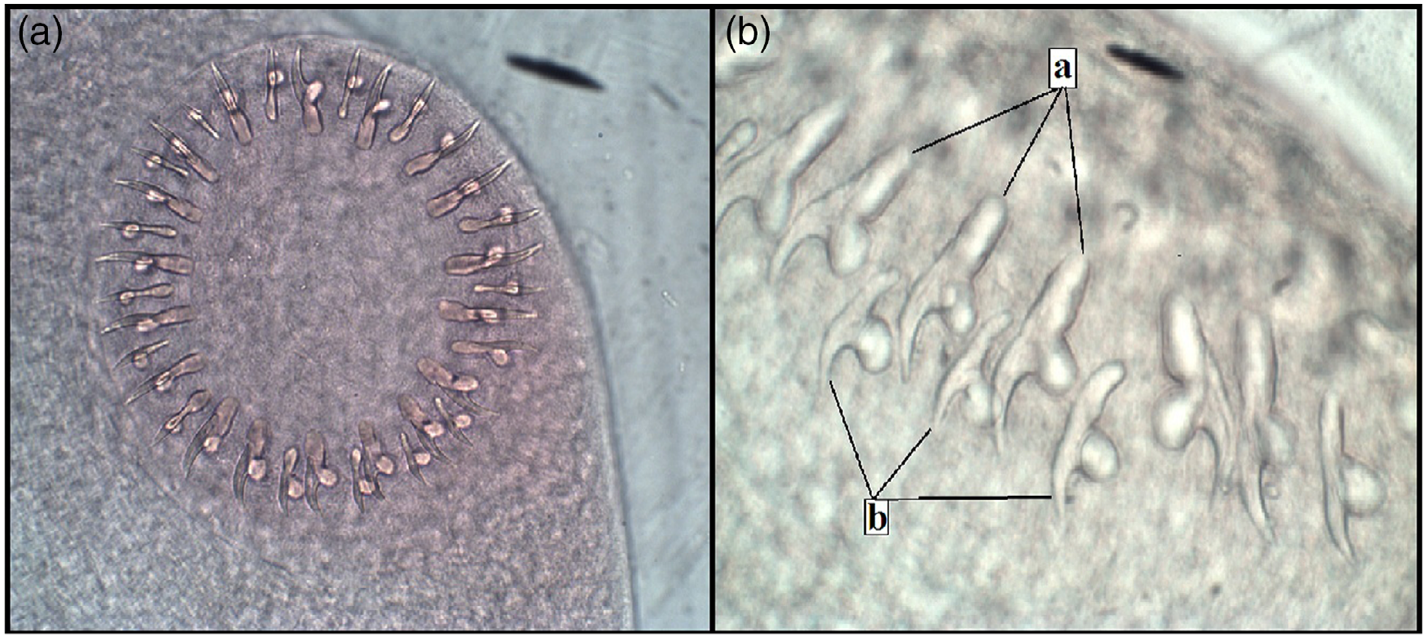
(a) Complete view of the morphological appearance of *Echinococcus granulosus* hooks and (b) large hooks (a) and small hooks (b).

Morphological examinations were carried out on these tapeworms. All tapeworms collected were recognized as *E. granulosus* based on their morphological characteristics. According to the images, these tapeworms had a branched uterus and a posteriorly inclined genital pore within the proglottid (Figure 2a,b) (Table 1). Measurements obtained in this study suggest that adult tapeworms in the northern and north‐western parts of Iran are considerably different in length (Figure 3a,b). The *Echinococcus* cestodes found in the north‐western region were longer than those found in the northern region. As indicated in Table 2, the mean total length of the adult tapeworms was 3.56 ± 0.2 mm, and the ratio of terminal proglottids as % to total length was 46.74 ± 0.95. The total number of rostellar hooks was 32.8 ± 1. Other measurements, such as the total length of the large and small hooks and the blade length of the large and small hooks, are shown in Table 2.

**TABLE 1:** The number and gender of wolves, jackals and fox carcasses in each of the three regions.

Animal	Region		
	Northeastern	Northern	Northwestern
Jackal	17 (8 F, 9 M)	35 (22 F, 13 M)	17 (10 F, 7 M)
Foxes	9 (6 F, 3 M)	0	13 (6 F, 7 M)
Wolfs	2 (1 F, 1 M)	0	0
Total carcasses	28	35	30

*Note*: F = female; M = male.

**TABLE 2:** Characteristics of rostellar hooks derived from *E.granulosus* in the present study compared to the previous studies.

	Present study				
	Geographical region			Thompson *et al.* (1984): Dog	Turceková et al. (1998): Dog	Shariatzadeh et al. (2015): Dog (G1)	Hosseini et al. (2012): Dog G1 (4)	Hosseini et al. (2012): Buffaloes G1	Thompson et al. (1984): Sheep	Gholami et al. (2011): Camel (Gholami et al., 2011)
Morphometrics	North of Iran	Northwest of Iran	Iran (Both of region)	Australia	Slovak Republic	North‐western Iran	North, northwest, southwest of Iran	North, northwest, southwest of Iran	Australia	North Iran
Total length (mm)	2.74 ± 0.1	4.38 ± 0.3	3.56 ± 0.2	2.5 ± 0.8	3.27 ± 0.45	‐	3.2 ± 0.7	‐	‐	‐
Gravid proglottid length (GPL) (mm)	1.30 ± 0.08	2.03 ± 0.08	1.66 ± 0.08	0.8 ± 0.3	1.63 ± 0.30	‐	1.7 ± 0.4	‐	‐	‐
Terminal proglottids as %/total length	47.2 ± 1.2	46.28 ± 0.7	46.74 ± 0.95	‐	48.3 ± 3.1	‐	44.2 ± 4.5	‐	‐	‐
**Number of hook**	33.1 ± 0.5	32.6 ± 1.5	32.8 ± 1	33.0 ± 3.4	31.0 ± 1.0	‐	33·3 ± 4.4	35·4 ± 1.7	35.0 ± 2.9	34.5
**Large hook**										
Total length (LTL) (µm)	34.1 ± 1.3	34.8 ± 0.5	34.45 ± 0.9	31.8 ± 2.5	34.9 ± 2.4	31.00 ± 3.21	31.6 ± 2	24.3 ± 1·2	25.01 ± 1.1	27 ± 1.6
Blade length (LBL) (µm)	11.8 ± 0.4	12.1 ± 0.2	11.95 ± 0.3	12.9 ± 1.0	12.7 ± 1.1	13.04 ± 0.75	13.1 ± 1.8	11.9 ± 1.4	12.4 ± 1.2	13.3 ± 1.4
LBL/LTL% (µm)	34.9 ± 1.2	34.9 ± 0.7	34.9 ± 0.9	40.1 ± 2.5	35.8 ± 3.1	42.04 ± 4.79	41.4 ± 2·5	49.0 ± 7·5	49.4 ± 4.5	48.2 ± 5.7
**Small hook**				‐	‐	‐	‐	‐	‐	‐
Total length (STL) (µm)	24.2 ± 0.5	25.5 ± 0.7	24.6 ± 0.5	24.6 ± 2.9	24.8 ± 2.7	22.28 ± 1.31	23.6 ± 1.7	20.1 ± 1.7	21.4 ± 1.5	22.6 ± 2.1
Blade length (SBL) (µm)	7.5 ± 0.3	8.1 ± 0.1	7.8 ± 0.2	9.1 ± 1.2	8.7 ± 1.0	7.48 ± 0.50	9.3 ± 0.8	8.6 ± 1.6	8.5 ± 0.9	9.6 ± 1.8
SBL/STL% (µm)	31 ± 1.1	32.1 ± 1.1	31.5 ± 1.1	37.8 ± 3.9	35.0 ± 3.6	33.8 ± 1.98	40.6 ± 2.6	42.8 ± 4.2	40.6 ± 3.5	43.4 ± 6.0

*Note*: Amin Pour *et al.* (2011), Gholami et al. (2011).

There is a significant difference between the total length of worms in the northern and north‐western regions (*p*‐value = 0.02). On the other hand, there was no significant difference between the length of the hooks of *Echinococcus* rostellum correlated with the geographical location (*p*‐value = 0.6626).

### 3.3 PCR‐RFLP results

**FIGURE 4:**
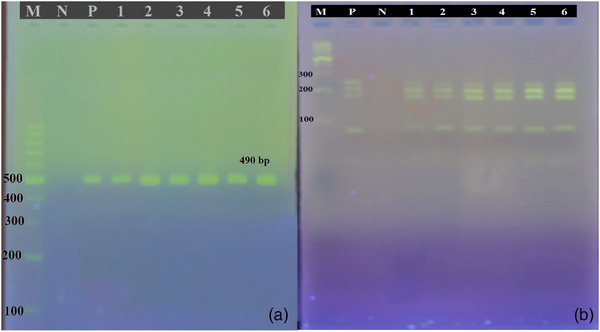
(a) Agarose gel electrophoresis of ITS1–polymerase chain reaction (PCR) products. M: DNA ladder (100 bp), N: negative control, P: positive control, north of Iran (1,2,3) and northwest of Iran (4,5,6). (b) Agarose gel electrophoresis of the PCR‐ITS1 products of *Echinococcus granulosus* isolate digested with the restriction enzyme Bsh1236I.

In PCR products generated in response to primers targeting the *ITS1* gene, a 490 base pair band was observed (Figure 4a). Four bands were detected when PCR‐RFLP was performed using the restriction enzyme Bsh1236I (BstUI) with *ITS1* gene PCR product. According to these results, this study determined the samples as *E. granulosus s.s*. (Figure 4b).

### 3.4 PCR and sequencing

#### 3.4.1 COXI

**FIGURE 5:**
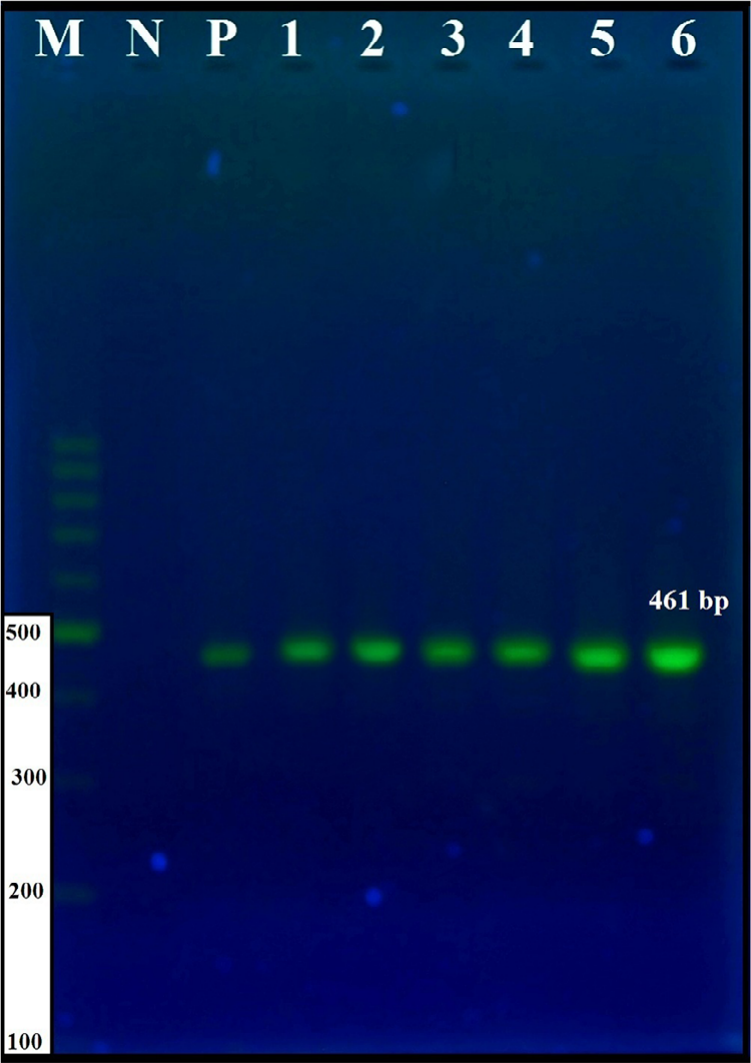
Agarose gel electrophoresis of Cox1–polymerase chain reaction (PCR) products. M: DNA ladder (100 bp), N: negative control, P: positive control, north of Iran (1,2,3) and northwest of Iran (4,5,6).

In all 42 samples, PCR products amplified based on primers specific for the *COXI* gene yielded a 460 base pair band (Figure 5).

The nucleotide sequence of a 460‐bp fragment of the COXI gene is different in three nucleotides between G1 and G2/G3. These three nucleotides are C/C/T in G1, and T/T/C in G2 and G3. In this study, most of the obtained sequences were the same as G1, but one sequence showed T/C/T. In a multiple alignment analysis of the COXI sequence, a nucleotide variation was observed in the isolates from north Iran. A transition (T instead of C nucleotide) occurred at position 95 in the nucleotide sequence. Although it may have been a nucleotide variation or PCR mistake, this did not affect the amino acid translation. Two sequences from the CoxI gene of samples collected from Iran's north and northwest regions were submitted as OR345478 and OR345479 in GeneBank. The sequencing results of the COXI PCR products showed 100% similarity to the registered sequences in the GenBank with accession numbers KX874711, EU178104, MT786855, HM598459, HF947559, MN990735, MT073987 and MW350099.

#### 3.4.2 NADH1

**FIGURE 6:**
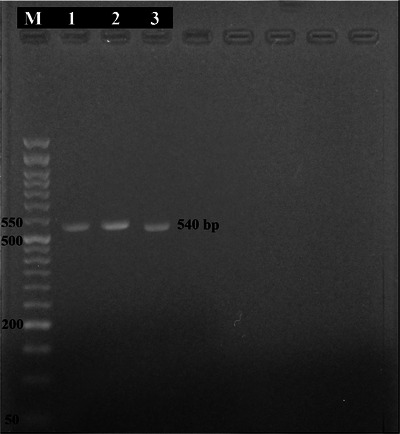
Agarose gel electrophoresis of NAD1–polymerase chain reaction (PCR) products. M: DNA Ladder (A: 100 bp and B: 50 bp), N: negative control, P: positive control, north of Iran (A) and northwest of Iran (B).

As a result of employing primers designed explicitly for the *NADH1* gene, 540 base pairs were amplified in the PCR products (Figure 6). The nucleotide sequence of a 540‐bp fragment of the *NADH1* gene is different in three nucleotides between G1 and G2/G3. These three nucleotides are A/C/A in G1 and G/T/G in G2 and G3. In the present study, all the obtained sequences were A/T/A. In a multiple alignment analysis of the *NADH1* sequence, a nucleotide variations (T/C) were observed between isolates from the north and northwest of Iran, but these variations did not affect the amino acid translation. Two sequences from the *NADH1* gene of samples collected from Iran's north and northwest regions were submitted as OR420756 and OR420757 in GeneBank.

The sequencing results of the *NADH1* PCR products showed 100% similarity to the registered sequences in the GenBank with accession numbers MH686293 and 99.41% to the KU925403, MG672170, MG672245, MG672267, MH050629, LC481609, LC481843 and DQ080021.

### 3.5 Phylogenetic analysis

The phylogenetic analysis based on the *CoxI* and *NADH1* sequence revealed six distinct clusters corresponding to different species and genotypes of *Echinococcus*. Within the G1 genotype, two divergent isolates from the north and northwest of Iran were identified in this study.

**FIGURE 7:**
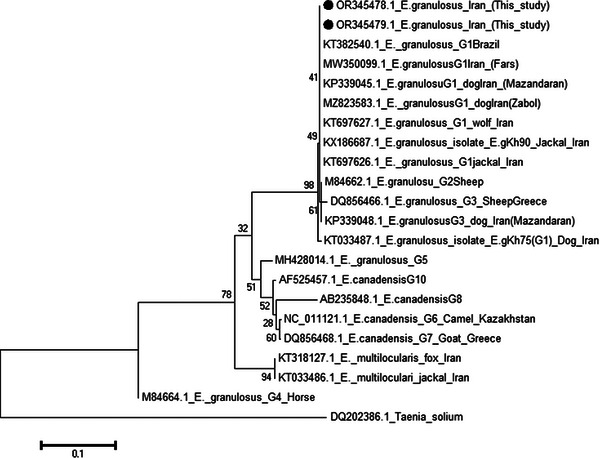
The phylogenetic tree of *Echinococcus granulosus* COXI derived in this study (showed with ●) and known *E. granulosus* genotypes and other species of *Echinococcus* in GenBank. The evolutionary analyses were conducted in MEGA11, and using the neighbour‐Joining method. The percentages of replicate trees in which the associated taxa clustered together in the bootstrap test (500 replicates) are shown next to the branches. The evolutionary distances were computed using the maximum composite likelihood method.

**FIGURE 8:**
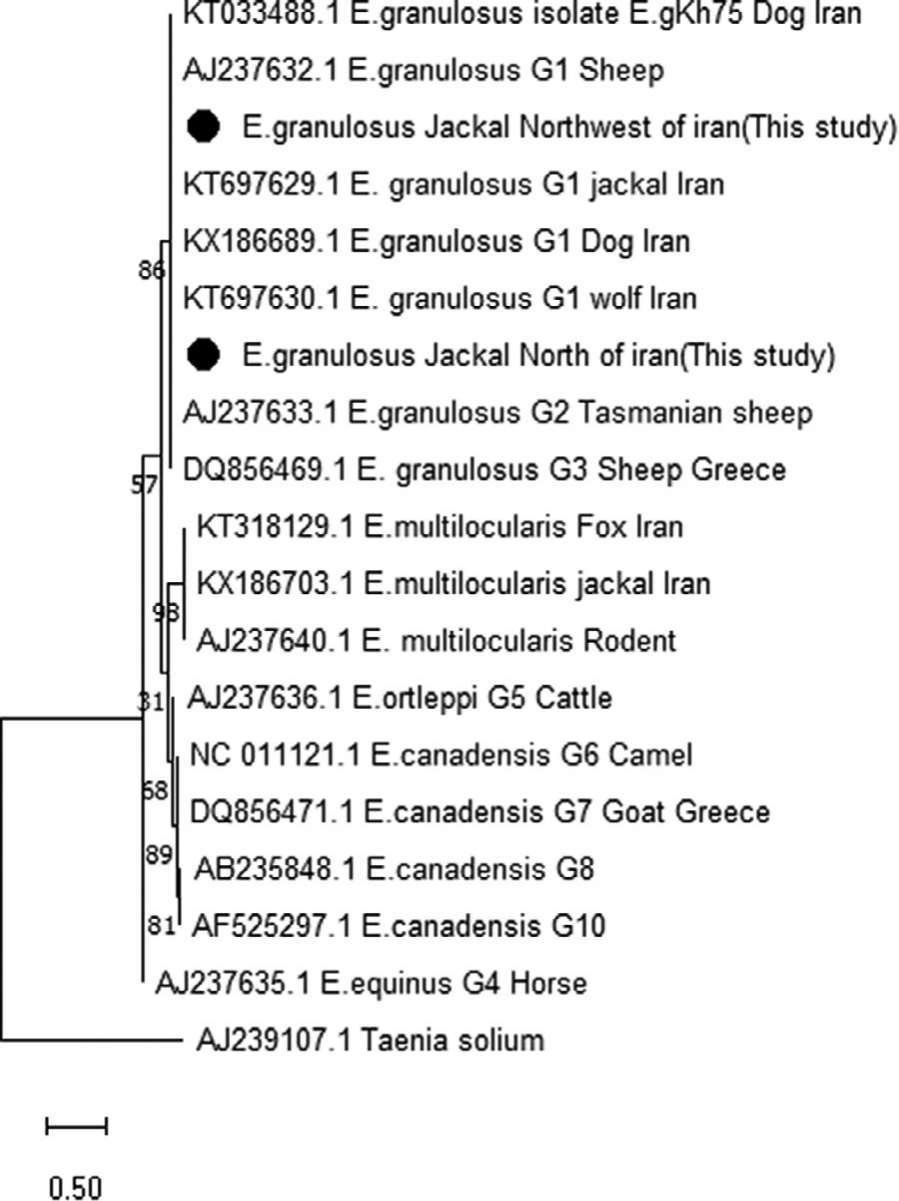
The phylogenetic tree of *Echinococcus granulosus* NADH1 derived in this study (showed with ●) and known *E. granulosus* genotypes and other species of *Echinococcus* in GenBank. The evolutionary analyses were conducted in MEGA11 using the neighbour‐Joining method. The percentages of replicate trees in which the associated taxa clustered together in the bootstrap test (500 replicates) are shown next to the branches. The evolutionary distances were computed using the maximum composite likelihood method.

Figure 1 shows the phylogenetic relationships of two representative isolates of Iran (north and northwest of Iran) obtained in this study, along with other G1 genotypes of *Echinococcus*. The Iranian isolates formed a monophyletic group closely related to the G2 and G3 genotypes. Other genotypes (G5, G6, G7, G8 and G10) (G6, G7, G8 and G10), and *E. ortleppi* (G5) also formed a distinct clade (Figures 7 and 8).

**FIGURE 9:**
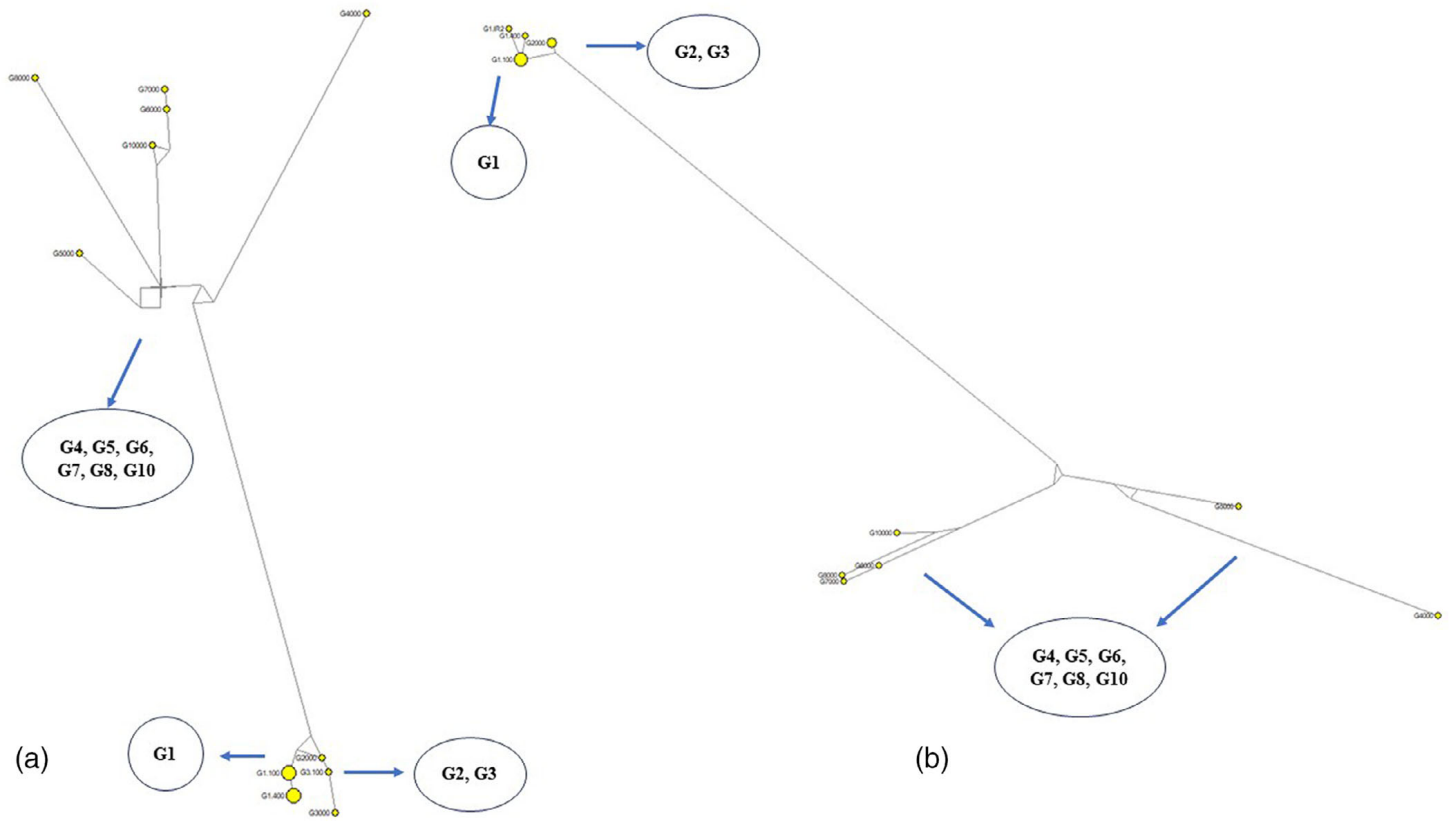
The phylogenetic network of *Echinococcus granulosus* CoxI and NADH1 derived in this study.

In the phylogenetic network, the CoxI and NADH1 gene fragments allowed a clear distinction between *E. granulosus s.s*. and *the other genotypes of E. granulosus s.l* Gholami et al. (2011), (Figure 9).

## 4 DISCUSSION

An investigation was performed in Iran's north, northwestern and northeastern regions to determine whether *E. granulosus s.l*. is prevalent among wild canids such as jackals, foxes and wolves. In the current study, *E. granulosus s.s*. was present in three (3.2%) samples. Infections with *E. granulosus s.s*. have only been found in jackals living close to human habitations. However, there is no infection in foxes and wolves. According to a previous report, the infection rate of *E. granulosus* in jackals in the northeast of Iran was 3.3%. However, the present study did not detect any infection in jackals from the same region, whereas the infection rate in jackals from two other regions (north and northwest of Iran) was consistent with the previous report (Heidari et al., 2019). Infection rates were lower than what previously was reported from the northeast of Iran (Beiromvand et al., 2011). Based on studies done in Iran's western provinces (Dalimi et al., 2002, 2006), it has been reported that jackals and foxes have different prevalence rates of *E. granulosus* with higher prevalence rates in foxes (4.54% vs. 2.3%). These amounts, in general, are also lower than what was reported from other West Asian countries such as Kazakhstan, South America with a prevalence of 66.03% (Avila et al., 2017; Cucher et al., 2016).

Differences in the prevalence of echinococcosis are not very clear. However, they may be because factors such as accessibility to intermediate hosts and environmental conditions, including climate and humidity, play a significant role (Thompson, 2023). Other factors like differences in environmental conditions conducive to the perpetuation of the parasite, abundance of infected definitive hosts, livestock husbandry, stocking rate, nature of the pasture and grazing patterns of animals may contribute to this variation (Grakh et al., 2020).

In the current study, the *Echinococcus* found in the jackals of northwest Iran exhibit a larger total length, with an average of 4.38 mm ± 0.3, compared to those found in the north of Iran, with a smaller average total length of 2.74 mm ± 0.1. In the comparison, our study reported a slightly higher total length than previous studies’ findings in dogs. In the present study, the total length of *Echinococcus* parasites in Iran (both regions) was 3.56 ± 0.2 mm. Comparing this with other studies conducted in dogs of Australia (2.5 ± 0.8 mm) and Slovakia (3.27 ± 0.45 mm), it is evident that the *Echinococcus* parasites in jackals of Iran exhibited a slightly larger total length (Turceková et al., 1998; Thompson et al., 1984). These intriguing findings underscore the complexities of host‐parasite relationships and open new avenues for further research into the ecological and biological factors influencing *Echinococcus* infections among diverse hosts in Iran. It is essential to note that despite the potential variability arising from different stages of parasite growth, the comparison conducted is based on an average of sizes. This discrepancy in size distribution could suggest variations in the developmental stages of *Echinococcus* within different geographical regions or populations of the definitive host. Moreover, it might reflect genetic diversity or adaptive mechanisms in response to environmental factors. Further investigation into these size differences could provide valuable insights into the biology and epidemiology of *Echinococcus* species, shedding light on factors influencing their transmission dynamics and pathogenicity.

The study compared hook sizes in *Echinococcus* parasites between different regions and host species in Iran and globally. The number of hooks was higher in the north than in the northwest. These findings highlight the complexities of host‐parasite interactions and call for further research on *Echinococcus* infections among diverse hosts in Iran.

The PCR‐RFLP‐ITS1 analysis of the *Echinococcus* samples in this study revealed four bands that matched the camel RFLP‐ITS1 pattern reported in Iran (Moghaddas et al., 2015). According to these results, this study determined the samples as *E. granulosus s.s*.

The nucleotide sequence of a 460‐bp fragment of the *COXI* gene is different in three nucleotides between G1 and G2/G3. These three nucleotides are C/C/T in G1 and T/T/C in G2 and G3 (Bowles et al., 1992). In this study, most of the obtained sequences were the same as G1, but one sequence showed T/C/T. However, it may be a nucleotide variation or PCR mistake.

The nucleotide sequence of a 540‐bp fragment of the *NADH1* gene is different in three nucleotides between G1 and G2/G3. These three nucleotides are A/C/A in G1 and G/T/G in G2 and G3 (Bowles & McManus, 1993). In the present study, all the obtained sequences were A/T/A. Considering that the two positions of the nucleotide are the same as G1, along with other data obtained from the *COXI* genes, the resulting sequences are most likely the same as G1.

The phylogenetic analysis based on the *CoxI* and *NADH1* sequence also revealed six distinct clusters corresponding to different species and genotypes of *Echinococcus*. The Iranian isolates formed a monophyletic group closely related to the G2 and G3 genotypes. As expected, *E. granulosus* (G4), *Echinococcus vogeli* and *Echinococcus oligarthrus* were placed in separate clusters. *Echinococcus multilocularis*, *E. canadensis* (G6, G7, G8 and G10) and *E. ortleppi* (G5) also formed a distinct clade.

Although the phylogenetic network analysis based on the *CoxI* and the *NADH1* gene fragments allowed separation between G1 and G2, this difference is not enough to show clear dissemination between *E. granulosus s.s*. strains/genotypes. Therefore, it is prudent to consider the species obtained in this study to be *E. granulosus s.s*. Additional studies, including the examination of more genes with longer lengths of PCR product, are needed to determine *E. granulosus s.s*. strains/genotypes.

Much less research has been conducted in Iran and worldwide on *E. granulosus s.l*. species/genotypes in the final host than in intermediate hosts. Until now, there is limited information on the molecular characterization of adult *E. granulosus s.l*. species/genotypes in golden jackals in Iran (Beiromvand et al., 2011; Heidari et al., 2019). Comparatively, the *E. granulosus* G1 genotype was reported previously in dogs in the northeast and west of Iran (Ghabdian et al., 2017; Parsa et al., 2012). Available molecular data from different livestock species, including sheep, goats, cattle and camels, revealing the existence of G1 genotypes in the northwest (Shariatzadeh et al., 2015), north (Gorgani‐Firouzjaee et al., 2019), northeast (Fadakar et al., 2015) and southwest (Fallahizadeh et al., 2019), indicated that *E. granulosus* G1 genotype was circulating in these regions. It has been reported that *E. granulosus* genotype G1 is most commonly found in intermediate and final hosts all over Iran (Hajimohammadi et al., 2022; Khademvatan et al., 2019).

These high similarities between the Iranian *E. granulosus* tapeworm sequences and those from different countries support the notion of global genetic connectivity among *E. granulosus* populations. This implies that *E. granulosus* might undergo international dispersion through various mechanisms, including animal migrations, human movement and the transportation of infected animals.

## 5 CONCLUSION

The results of this study offer valuable insight into *E*. granulosus prevalence and genotypic distribution among Iranian wild canid populations. The results confirm echinococcosis’ endemic status in Iran and demonstrate the significance of regional differences in prevalence rates and genotypic composition. The detection of *E. granulosus s.s*. strains/genotypes and the observed nucleotide variations highlight the importance of continued surveillance and monitoring of this zoonotic parasite. Furthermore, the high sequence similarity with isolates from different countries reinforces the need for international collaboration in addressing the challenges posed by *E. granulosus* and devising effective control and prevention strategies. The research findings emphasize the need for further surveillance, control measures and public awareness campaigns.

## AUTHOR CONTRIBUTIONS

**Moein Abolhasani Darounkola**: Data curation; formal analysis; investigation; methodology; software; writing—original draft; writing—review and editing. **Elahe Ebrahimzadeh**: Conceptualization; data curation; investigation; methodology; project administration; resources; software; supervision; validation; visualization; writing—original draft; writing—review and editing. **Hassan Borji**: Conceptualization; data curation; investigation; methodology; resources; validation; visualization; writing—review and editing. **Mohammadreza Khoshvaght**: Data curation; methodology; writing—original draft. All authors checked and approved the final version of the manuscript for publication in the present journal.

## CONFLICT OF INTEREST STATEMENT

The authors declare no conflicts of interest.

### ETHICS STATEMENT

All applicable international, national and/or institutional guidelines for the care and use of animals were followed (Ethical code: IR.UM.REC.1401.108).

### PEER REVIEW

The peer review history for this article is available at https://publons.com/publon/10.1002/vms3.1554.

## Data Availability

The datasets generated during and/or analysed during the current study are available from the corresponding author on reasonable request.
